# Changes in the Erythrogram Parameters and in the Erythrocyte Sizes of Adult *Pelophylax ridibundus* (Pallas 1771) (Anura: Ranidae) Inhabiting the Sedimentation Lake of Brikel Thermal Power Plant in Southern Bulgaria

**DOI:** 10.3390/toxics13040261

**Published:** 2025-03-29

**Authors:** Zhivko Zhelev, Tihomir Vachev, Danail Minchev

**Affiliations:** 1 Department of Human Anatomy and Physiology, Faculty of Biology, Paisii Hilendarski University of Plovdiv, 24 Tsar Asen Str., 4000 Plovdiv, Bulgaria; 2 Department of Molecular Biology, Faculty of Biology, Paisii Hilendarski University of Plovdiv, 24 Tsar Asen Str., 4000 Plovdiv, Bulgaria; tihomirvachev@uni-plovdiv.bg

**Keywords:** marsh frogs, environmental pollution, red blood cells, erythrogram, erythrocyte-metric parameters, ecotoxicology

## Abstract

Analyses of the hematological statuses of animals inhabiting areas of anthropogenic pollution may provide valuable insights into the extent of disturbance of their living conditions and the mechanisms of adaptation to various environmental stressors. The current work compares the erythrogram and erythrocyte-metric parameters of marsh frogs (*Pelophylax ridibundus*) inhabiting the polluted sedimentation lake of Brikel TPP in southern Bulgaria to those in frogs inhabiting a relatively clean habitat (reference population). The study includes a total of 120 individuals (30 females and 30 males from each site). For all of them, total erythrocyte count, hemoglobin concentration, hematocrit, MCV, MCH, and MCHC were evaluated via standard laboratory techniques. All erythrocyte metrics were determined microscopically in two blood smears from each animal. Our study reveals alterations in the erythrogram parameters and the erythrocyte sizes in marsh frogs living in conditions of chronic pollution with industrial wastewater compared to the animals from the reference site. The mean values for all erythrocyte morphology parameters are significantly lower in both female and male individuals inhabiting the polluted area compared to those originating from the reference one. Conversely, three erythrogram parameters—erythrocyte count, hemoglobin, and hematocrit—appeared significantly higher in females and males from the polluted site. The observed changes in the erythrogram parameters and erythrocyte sizes result from the deteriorated water quality of the sedimentation lake.

## 1. Introduction

Analyses performed immediately after capture, often in natural conditions (in situ), are informative approaches for assessing the health status of various animal populations living under specific environmental conditions [1]. Studying the chronic impact of toxicants on the physiology of test animals provides an objective overview of the state of their living environment. Furthermore, it reveals mechanisms and levels of adaptation to environmental stressors, including those of anthropogenic origin [2,3].

Because of their semi-permeable skin, adult aquatic frogs (Amphibia: Anura) are in direct contact with various toxicants [4]. This semi-permeability allows the bodies of aquatic frogs to balance between the following two environments: land and water. However, it also increases the risk of exposure to various toxic agents and creates a potential route for their penetration and absorption through bio-membranes [5]. Amphibians develop specific feeding habits during the different stages of their life (detritophagous or active predators). In the larval stages, they rely on the continuous water flow through the gills. Their metabolism is heterothermic, and they show remarkable sensitivity to chemicals during their freshwater cycles. All these unique features make anuran amphibians suitable bioindicators of general environmental conditions [6,7,8].

Despite the global decline in amphibian populations on every continent, there are also amphibians that not only manage to adapt and survive in stressful conditions but even form abundant populations in habitats affected by anthropogenic pollution [9]. In Eurasia, such a species is the marsh frog (*Pelophylax ridibundus*). This anuran species is suitable as a test object in in situ studies, as it responds to environmental stress through changes in various morphological and physiological traits that can be evaluated quantitatively [10,11,12]. Assessment of the morphophysiological biomarkers of *P. ridibundus* in habitats affected by anthropogenic pollution complements the data obtained from the chemical monitoring of water bodies. It offers an integrated assessment of the impact of pollutants on the general health status of marsh frogs [13,14].

In our previous work [15], we presented the leukogram parameters of adult *P. ridibundus* inhabiting the polluted sedimentation lake of Brikel TPP in southern Bulgaria, compared with marsh frogs inhabiting a relatively clean habitat (reference population). The present work aims to investigate the erythrogram parameters and the sizes of erythrocytes of *P. ridibundus* individuals from the same populations. In the course of the study, we tested a working hypothesis based on the assumption that changes in the erythrogram and the size of the erythrocytes can be expected (of an adaptive or pathological nature) in frogs living under conditions of chronic industrial wastewater pollution compared to those in the reference site.

## 2. Materials and Methods

### 2.1. Sampling Area and Data Collection

**Figure 1:**
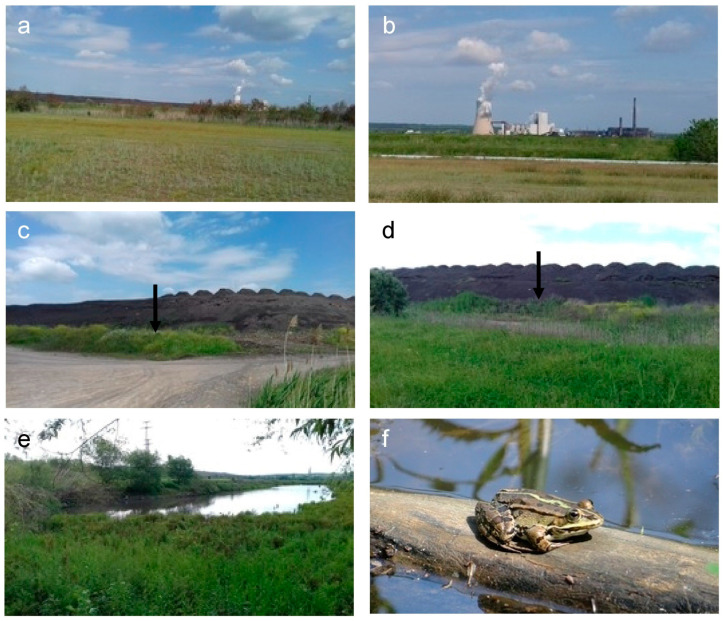
(**a**) General view of the sedimentation lake; (**b**) TPP AES “Galabovo” and TPP “Brikel”; (**c**,**d**) heaps of industrial ash; (**e**) a temporary body of water within the area of the sedimentation lake; (**f**) a male individual of *Pelophylax ridibundus*. Places where frogs were captured (**arrows**, **e**).

This study was conducted during the breeding season of *P. ridibundus* in April at two sites in southern Bulgaria. The polluted site (PS) was the sedimentation lake of Brikel TPP, near the town of Galabovo. The chosen reference site (RS) was the less-disturbed Vacha River, near the town of Krichim. A detailed physical–geographical map with coordinates of both locations can be found in [15]. The sedimentation lake of Brikel TPP is located northwest of the town of Galabovo, near Brikel TPP and the newly built TPP AES Galabovo, commissioned in 2011 (Figure 1).

The water used in the production cycles of the two thermal plants comes from the neighboring Rozov Kladenets Dam Lake. In recent years, the water in this artificial reservoir comes mainly from the Sazliyka River and, to a lesser extent, from the Sokolitsa River [16]. Because of the specific nature of the technological process of sedimentation of industrial ashes in different parts of the sedimentation lake, there are areas with water all year round. The central part of the slag dump (the industrial ash mounds), where the high-temperature water from the TPPs enters the lake, is practically unsuitable for living organisms. However, in the area between the slag dump and the outer embankments of the lake there are conditions for the development of diverse biota, including tailless amphibians (see Figure 1). In the present study, the sedimentation lake of Brikel TPP is considered a site polluted by anthropogenic influence. The reference site in the present study (site 2: Vacha River) is located south of the town of Krichim in an area devoid of anthropogenic activity. Detailed data from the physicochemical monitoring of water in Rozov Kladenets Dam Lake and the Vacha River carried out at the time of the study by the Basin Directorate for Water Management—East-Aegean Sea, Region Plovdiv, Ministry of the Environment and Water (https://earbd.bg), can be found in [15].

### 2.2. Physicochemical Properties of the Water at the Two Sampling Sites

The physicochemical parameters of waters from both sampling sites are cited in our previous paper [15] and provided here as a separate table (Table 1). Physicochemical data of the sedimentation lake of the Brikel TPP in 2019 suggest a significantly deteriorated water quality (Table 1). Oxidized and non-oxidized nitrogen, including nitrate nitrogen (NO_3_^−^-N), nitrite nitrogen (NO_2_^−^-N), ammonium nitrogen (NH_4_^+^-N), and total nitrogen (TN), exceed the permissible concentrations instilled by Ordinance No. H-4 of 14.09.2012 in Bulgaria [17]. The presence of abnormal concentrations of nitrite nitrogen is especially concerning since high levels of nitrites pose a significant health risk [18]. The water from the sedimentation lake is excessively eutrophic, having high concentrations of orthophosphates (PO_4_^3−^) and total phosphorus (P). It shows mildly deteriorated oxygen content in terms of low oxygenation (Ox) and biological oxygen demand (BOD5) near the upper margin of the norms. A water basin with such characteristics seems to exert versatile effects on the status of anuran amphibians in the area. From one perspective, the sedimentation lake provides a relatively secluded habitat for marsh frogs to inhabit undisturbed. However, the abnormal physicochemical properties of its water negatively impact its living conditions.

### 2.3. Blood Tests and Data Analysis

The analyses of the erythrogram and erythrocyte-metric parameters of the frogs were conducted with samples of equal size—30 male and 30 female individuals (adults with SVL > 50.0 mm, following Pile et al. [19]) from each site. Marsh frogs were captured in the water at night using a flashlight [15]. To minimize the stress, animals were transferred in buckets with water from the capture site to the laboratory. Blood for the tests was collected from the heart with a small heparinized needle (Fisher Scientific, Pittsburgh, PA, USA) following anesthesia with diethyl ether. Capturing and blood sampling were carried out in accordance with Directive 2010/63/EU of the European Parliament. Blood samples were stored in heparinized tubes and analyzed in a laboratory setting. After the manipulations, the frogs were released into their natural habitat. The erythrogram parameters included total red blood cell count (RBC), hemoglobin concentration (Hb), and packed cell volume (PCV) and were determined using standard methods (counting erythrocytes with a Burker chamber, cyanohemoglobin method, and microhematocrit method, respectively), described in detail by Ware et al. [20]. The hematological indices, MCV, MCH, and MCHC, were calculated using mathematical formulas according to Ware et al. [20] and Wahed and Dasgupta [21]. Erythrocyte sizes were determined in two blood smears for each individual following Giemsa–Romanowsky staining (Merck KGaA, Darmstadt, Germany) with an Olympus SZX16 stereo microscope (Olympus, Shinjuku City, Tokyo, Japan). Precisely 40 (20 per smear) randomly selected erythrocytes from each individual were measured with an ocular micrometer (MOB-1-15x) according to Arserim and Mermer [22] and Zhelev et al. [23,24]. The following four main erythrocyte dimensions were measured: erythrocyte length (EL), erythrocyte width (EW), nucleus length (NL), and nucleus width (NW). The shapes of the cells and nuclei were assessed by the ratios EL/EW and NL/NW and the cytonuclear ratio by NS/ES. We used Formulas (1) and (2) to calculate the surface area of the erythrocytes (ES µm^2^) and their nuclei (NS µm^2^), according to Arserim and Mermer [22] and Zhelev et al. [23,24].

ES = EL × EW × π/4(1)

NS = NL × NW × π/4(2)

Statistical processing was conducted using the R statistical language (version 4.0.3). The experimental data were checked for normality with the Shapiro–Wilk test. A Kaiser–Meyer–Olkin (KMO) test was applied to verify the suitability of the obtained data for multifactorial analysis. The multifactorial analysis was performed as a principal component analysis (PCA). This method recasts the original dataset to reduce the number of variables and, thus, to outline variables with significant correlation. The choice of principal components was made based on Kaiser’s criterion (eigenvalues >1) and visual examination of a scree plot. A two-way ANOVA method was applied to assess the effects of site and sex on the examined random variables. A Tukey HSD post hoc test evaluated the statistical significance of the observed differences among the examined groups. The homogeneity of variances was checked via Levene’s test.

## 3. Results

### 3.1. Kaiser–Meyer–Olkin (KMO) Test

A standard Kaiser–Meyer–Olkin (KMO) test was applied to determine the suitability of the obtained data for factor analysis. The overall KMO value for the examined dataset was ‘meritorious’ (0.803), which defines the experimental data as probably suitable for factor analysis. The individual KMO values for each separate variable were as follows: EL (0.802), EW (0.790), EL/EW (0.859), ES (0.808), NL (0.848), NW (0.783), NL/NW (0.776), NS (0.897), NS/ES (0.846), RBC (0.927), Hb (0.787), PCV (0.790), MCH (0.497), MCHC (0.142), and MCV (0.598).

### 3.2. Principal Component Analysis (PCA)

**Figure 2:**
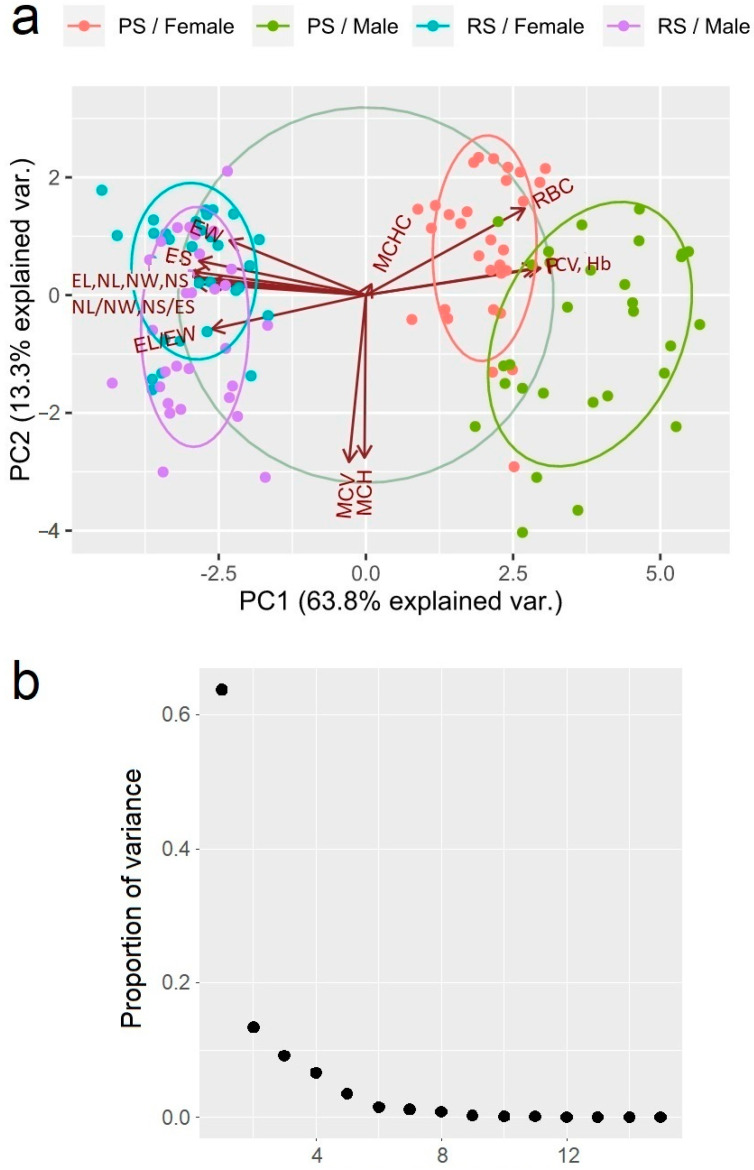
(**a**) A diagram of the correlations of erythrogram and erythrocyte-metric parameters in the *Pelophylax ridibundus* individuals from the investigated sites with the first two main axes. The distinguishing force of the parameter is indicated by the arrow length; a large importance is shown by the long arrow and it is strongly correlated with the ordination axes. (**b**) A scree plot of the proportion of variance explained by each principal component.

The results from the principal component analysis (PCA) identified three major principal components (PC1, PC2, and PC3) that explain 63.7% (eigenvalue = 9.564), 13.3% (eigenvalue = 2.001), and 9.1% (eigenvalue = 1.368) of the total variance, respectively (Figure 2). Three of the examined parameters—RBC (0.273), Hb (0.300), and PCV (0.291)—showed a moderate positive correlation with the first principal component, while the following nine others were negatively correlated with it: EL (−0.306), EW (−0.235), EL/EW (−0.265), ES (−0.287), NL (−0.317), NW (−0.296), NL/NW (−0.294), NS (−0.314), and NS/ES (−0.269). The following two parameters showed a strong negative correlation with the following second principal components: MCH (−0.611) and MCV (−0.628). Intriguingly, the MCHC parameter was the only one showing a positive correlation with the third principal component (0.779).

### 3.3. ANOVA

A two-way ANOVA was applied to evaluate the effect of site and sex on each of the examined hematological parameters. The results of the two-way ANOVA revealed a significant interaction effect between site and sex on the following erythrocyte metrices: EL (F(1, 116) = 28.3, *p* < 4.93 × 10^−7^), EW (F(1, 116) = 23.4, *p* < 3.98 × 10^−6^), ES (F(1, 116) = 22.8, *p* < 5.11 × 10^−6^), NL (F(1, 116) = 31.1, *p* < 1.57 × 10^−7^), NW (F(1, 116) = 48.3, *p* < 2.26 × 10^−10^), NS (F(1, 116) = 40.6, *p* < 3.84 × 10^−9^), and NS/ES (F(1, 116) = 7.7, *p* = 0.006). Conversely, this statistical analysis found no significant site-sex interaction effect on RBC (F(1, 116) = 0.1, *p* = 0.793), Hb (F(1, 116) = 0.4, *p* = 0.487), PCV (F(1, 116) = 1.0, *p* = 0.307), MCH (F(1, 116) = 0.6, *p* = 0.418), MCHC (F(1, 116) = 2.8, *p* = 0.093), MCV (F(1, 116) = 1.2, *p* = 0.265), EL/EW (F(1, 116) = 2.2, *p* = 0.14), and NL/NW (F(1, 116) = 0.5, *p* = 0.448).

The analysis of the simple main effects suggested that the site exhibits a strong statistically significant effect (*p* < 2 × 10^−16^) on all examined parameters, except on MCH, MCHC, and MCV (*p* > 0.05). Meanwhile, sex showed a statistically significant simple main effect on EL, EW, EL/EW, ES, NL, NW, NL/NW, NS, RBC, and MCV (Table 2).

The results from the post hoc Tukey HSD test demonstrated that the mean values for EL, EW, EL/EW, ES, NL, NW, NL/NW, NS, and NS/ES are significantly lower in both female and male individuals inhabiting the polluted site compared to those originating from the reference site. Conversely, three other parameters—RBC, Hb, and PCV—appeared significantly higher in both females and males from the polluted site (Table 3).

Overall, the red blood cell parameters and sizes of the frogs from the polluted sedimentation lake suggest erythrocytosis and increased hemoglobin and hematocrit levels, as well as shortening and rounding of RBCs of both sexes.

## 4. Discussion

**Figure 3:**
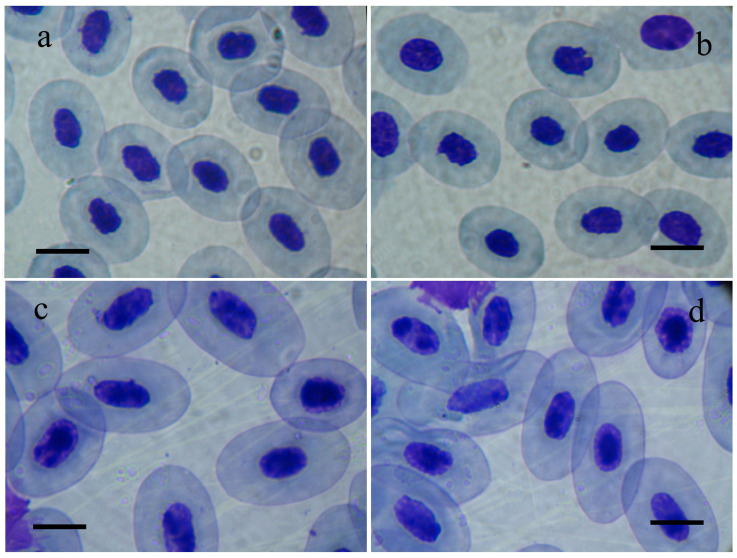
Photomicrographs of erythrocytes of *Pelophylax ridibundus* individuals from the polluted site, (**a**,**b**) the sedimentation lake of Brikel TPP, and the reference site, (**c**,**d**) Vacha River in southern Bulgaria. Scale lines = 10 μm.

The results of the present paper build upon our previous study of the sedimentation lake of Brikel TPP as a site of anthropogenic pollution. Our present study provides further details on hematological changes in marsh frogs inhabiting that specific polluted area. The results of this study establish statistically significant erythrocytosis, hyperchromia, and high hematocrit values of *P. ridibundus* individuals that inhabit the sedimentation lake of Brikel TPP compared to frogs from the reference population. These observations suggest a typical adaptive response of the erythrogram of sedimentation lake frogs to the stressful agents present in the environment (mainly large amounts of industrial ash) [16,25]. According to the general adaptation syndrome theory, the response against stress develops in the following three stages: alarming reaction, resistance stage, and exhaustion stage. The latter two show the pathological effects of prolonged exposure to stressors [26]. The alarm reaction develops under acute stress and is represented by the fight-or-flight response of the organism. Under chronic stress conditions, the body enters the resistance stage when developing adaptations to cope with higher stress levels [27]. If the prolonged stressor exists further, the organism enters the exhaustion stage, which pushes the body to its limit of physical coping with the stress conditions. Thus, our results prompt us to assume that the changes in erythrogram parameters and erythrocyte sizes can potentially reflect either adaptation or terminal exhaustion of the organism. In this case, the actual nature of the observed alterations (adaptive or exhaustive) will depend on the lasting impact of the anthropogenic stress and the exact phase of the response against it. Our results provide a rationale for the assumption that marsh frogs inhabiting the polluted site struggle to overcome the lasting damaging effects of the toxic substances, mainly industrial ash, in their environment. Given the higher RBCs, hemoglobin, and hematocrit in the animals from the polluted site, we can presume the adaptive role of the observed alterations. In polluted waters, erythrocytosis, hyperchromia, and high hematocrit facilitate the exchange and transport of oxygen and carbon dioxide, which allows the animals to resist the deleterious effects of pollutants. Elevated hemoglobin and hematocrit levels can result from a chronic decrease in plasma volume, often termed stress erythrocytosis [28]. Changes in erythrogram parameters and erythrocyte sizes have been reported for populations of frogs living under conditions of anthropogenic stress [29,30]. The results of the ANOVA analysis showed significant variations in erythrocyte morphology in addition to the observed differences in the total erythrocyte count, hemoglobin concentration, and erythrocyte volume percentage. The reason is that lower EL, EW, and EL/EW values indicate a more circular rather than elliptical shape of erythrocytes. Since lower EL, EW, and EL/EW are predominantly found in animals from the polluted site, we can claim that their erythrocytes would appear smaller and more circular (Figure 3).

The observed differences in the RBC sizes reflect a smaller, more circular shape of erythrocytes. RBCs become smaller as they age [31]. However, the potential adaptive role of such morphology can be due to compact erythrocytes allowing a fast release of oxygen in tissues with high metabolic demands [32]. According to a previous study by the team, the dynamics of RBC sizes in marsh frogs correlate with the nature of pollutants, their concentrations, and, to a lesser extent, the type of water basin. Previous studies by the team have established that EL, EW, and ES increase in the case of sewage pollution, while NL, NW, and NS remain unaffected. On the contrary, in the presence of heavy metals, the sizes EL, EW, ES, NL, NW, and NS appear significantly reduced [33]. The nucleocytoplasmic ratio (NS/ES) decreases in marsh frogs living in anthropogenic pollution, regardless of the type of toxicants, being more pronounced in water basins polluted with heavy metals [33].

Intriguingly, the present study suggests no significant between-group differences for the MHC, MCHC, and MCV parameters. Despite the changes in erythrocyte metrics, the MCV, MCH, and MCHC remain indifferent between the examined groups. It shows that RBCs of PS frogs undergo alterations in their shapes and sizes rather than in their volume or hemoglobin content. According to Johnstone et al. [34], temporary stress-related alterations in Hb and PCV are associated predominantly with fluctuations in plasma volume rather than in erythrocytes alone. Therefore, RBC-specific parameters, such as MCV or MCHC, are unlikely to be affected by short-term stressors.

Such morphological differences together with the significantly higher erythrocyte counts, hemoglobin levels, and hematocrit percentages in the animals from the polluted site may be discussed as an adaptive response to anthropogenic stress [16,25,30]. The higher RBC, hemoglobin, and hematocrit may potentially reflect increased oxygen demands in the animals from the polluted site. In this context, smaller compact erythrocytes with a more circular shape may provide improved oxygen exchange combined with increased erythrocyte and hemoglobin levels. Similar adaptive changes in the size and shape of erythrocytes, accompanied by a large number of erythrocytes and high hemoglobin in the peripheral blood of *P. ridibundus* have been reported by Zhelev et al. [23,33] and Dönmez and Şişman [13]. Intriguingly, our previous work on *P. ridibundus* conducted in the region of the Plovdiv non-ferrous metals plant reported partially dissimilar results [24]. In the blood of *P. ridibundus* inhabiting this contaminated site, there were small erythrocytes of a similar shape. However, there were also statistically significant (compared to the reference group) erythropenia and hypochromia, suggesting no adaptive biological response in these frogs. Although the leucogram parameters showed a markedly deteriorated immune status in the frogs from the sedimentation lake of Brikel TPP [15], the changes in the erythrogram and erythrocyte metric parameters of *P. ridibundus* individuals give us reasons to believe that the frogs manage not only to survive but also to adapt to life in this site heavily influenced by industrial anthropogenic activity.

Another interesting feature of the results from the Tukey HSD test is that the morphological parameters EL, NL, and NS/ES exhibit statistically significant differences between sexes only in individuals inhabiting the polluted site (see Table 3 and Figure 2). Overall, the mean values of EL, NL, and NS/ES are markedly lower in males inhabiting the polluted site than in females from the same site. Conversely, no significant sex-based difference was observed in the same parameters when comparing females and males from the reference site. These experimental data suggest that female and male individuals may exhibit different adaptive capabilities under anthropogenic stress. However, further analysis with more diverse biomarkers is needed to confirm this assumption.

The applied PCA differentiates the animals inhabiting the sedimentation lake from those from the less disturbed habitat. Together with the ANOVA analysis, this leads to the assumption that the population of marsh frogs inhabiting the sedimentation lake of Brikel TPP and those in the less disturbed area have contrasting physiological statuses. Notably, the parameters showing correlation with the first principal component differ significantly among the examined groups of animals. The PCA of erythrogram and erythrocyte-metric data correctly segregate the animals from both habitats. Thus, higher erythrocyte count, hemoglobin, and hematocrit combined with lower erythrocyte-metric parameters may reflect the animal’s fitness under environmental stressors when animals are more likely to experience increased metabolic demands [35,36]. They trigger stimulation of the hemopoietic organs and lead to an increasing number of blood cells involved in gas exchange and transport [33]. The increased hemoglobin synthesis leads to a higher hemoglobin concentration in the blood. Furthermore, smaller cells can provide faster gas delivery in tissues [37] and improved oxygen saturation in hypoxic conditions [38]. Considering these assumptions, we can suspect that the changes in RBC parameters in frogs from the sedimentation lake might improve body resistance and are more likely adaptive than exhaustive.

Meanwhile, the same PCA separates male from female individuals in the polluted area. This intriguing piece of evidence, alongside the above findings, suggests that male individuals suffer more considerable damage than females when living under conditions of anthropogenic stress. That is another interesting observation, which implies that male marsh frogs are more severely affected than females when living under conditions of anthropogenic pollution. Probably, male individuals experience a more stringent evolutionary selection and, thus, are eliminated selectively and faster than females. From a biological perspective, this stringent selection is justified, since it lessens the impact of the deteriorated environment on the reproductive potential of the population [39,40].

Future studies are still required to further examine the effect of anthropogenic stress on the population of P. ridibundus inhabiting the sedimentation lake. Our results again demonstrate that the in situ analyses of hematological parameters give adequate and informative insights into the physiological status of anuran amphibians inhabiting areas of anthropogenic pollution. As might be expected, this type of study has its limitations. For instance, a longitudinal evaluation of the examined erythrogram and erythrocyte-metric parameters can provide a better understanding of their seasonal dynamics. The most significant limitation of the present paper is that it does not provide a mechanistic explanation of the association between hematological data and the specific effects of pollutants. The evaluation of the specific impacts of environmental toxicants at molecular and biochemical levels is out of the scope of the present study. Revealing the intricate causal mechanisms that trigger the observed erythrogram and erythrocyte-metric changes require more precise biochemical, molecular, and genetic analyses. Although they do not answer all questions, in situ studies conducted with informative biomarkers can be used as an early diagnostic tool to identify problems in animal populations living under environmental stress conditions.

## 5. Conclusions

In this research, we confirmed erythrocytosis, hyperchromia, and high hematocrit values in the blood of *P. ridibundus* individuals that inhabit the sedimentation lake of Brikel TPP, comparing them with frogs from the reference site. We suspect that these alterations in the erythrogram are adaptive changes in response to stress. The statistical analysis of the simple main effects reveals that the site exhibits a strong statistically significant effect on all examined parameters, except on hematological indices (MCH, MCHC, and MCV). Our experimental data also demonstrate that the mean values for EL, EW, EL/EW, ES, NL, NW, NL/NW, NS, and NS/ES are significantly lower in both female and male individuals inhabiting the polluted site compared to those originating from the reference one. In our opinion, the above changes in the erythrogram and erythrocyte sizes result from the deteriorated water quality of the sedimentation lake because it contains toxicants and industrial ash. Our results provide a rationale for the assumption that marsh frogs inhabiting the polluted sedimentation lake struggle to overcome the lasting damaging effects of the toxic substances in their environment. They bring risks for the exhaustion and decline of the examined population in the future, provided that pollution still exists.

The most significant limitation of the present paper is that it does not provide a mechanistic explanation of the association between hematological data and the specific effects of pollutants. Further efforts are still required for a better understanding of the seasonal dynamics of the examined erythrogram and erythrocyte-metric parameters.

## Figures and Tables

**Table 1:** Physicochemical parameters of waters from the sedimentation lake of Brikel TPP/Rozov Kladenets Reservoir and the Vacha River (adapted from [15]; data are provided by the Basin Directorate for Water Management—East-Aegean Sea, Region Plovdiv, Ministry of the Environment and Water (https://earbd.bg)).

Parameters and Standards for High Water Quality April 2019		Reference Site: Vacha River, Near the Town of Krichim		Polluted Site: Sedimentation Lake of Brikel TPP/Rozov Kladenets Reservoir	
		Average Annual for 2019	April 2019	Average Annual for 2019	April 2019
Temperature °C	–	12.8	–	19.0	–
Ph units	–	8.0	8.1	8.1	8.3
EC µS/cm	700.0	201.0	192.6	1088.0	994.0
DO mgO_2_/L	9.0–7.0	10.4	10.1	7.9	7.8
BOD5 mgO_2_/L	<2.0	1.0	0.8	2.4	1.9
COD mgO_2_/L	25.0	9.0	–	23.0	–
Ox %	100–105	117.0	–	88.0	–
NH^4+^-N mg/L	<0.10	0.04	0.08	0.35	0.53
NO_2_^−^-N mg/L	<0.03	0.01	0.01	0.08	0.14
NO_3_^−^-N mg/L	<0.7	0.57	0.55	1.14	1.12
TN mg/L	<0.7	<1.0	<1.0	2.5	2.4
PO_4_^3−^ mg/L	<0.07	0.04	0.06	0.25	0.34
TP mg/L	<0.15	0.01	0.02	0.27	0.45

Legend: EC—electrical conductivity, DO—dissolved oxygen, Ox—oxygenation, BOD5—biological oxygen demand five days, COD—chemical oxygen demand, NH^4+^ -N—ammonium nitrogen, NO_2_^−^ -N—nitrite nitrogen, NO_3_^−^ -N—nitrate nitrogen, TN—total nitrogen, PO_4_^3−^—orthophosphates, TP—total phosphorus. Standards for water quality are instilled by Ordinance № H-4 of 14.09.2012 (State Gazette, № 22. 5.03.2013) [17] on the characterization of surface waters in Bulgaria.

**Table 2:** Two-way ANOVA: tests of the between-subjects effects (Adf: Sites, Bdf: Sex, Cdf: Sites x Sex, Ddf: Residuals; and MS: Mean square) for erythrogram and erythrocyte metric parameters in *Pelophylax ridibundus* individuals from two sites in southern Bulgaria.

**RBC**	**Hb**	**PCV**	**MCH**	**MCHC**	**MCV**	**EL**	**EW**
A_1_: MS = 1,845,120	A_1_: MS = 26963	A_1_: MS = 0.5175	A_1_: MS = 16.5	A_1_: MS = 79.5	A_1_: MS = 3589	A_1_: MS = 164.56	A_1_: MS = 11.501
F = 380.4 ***	F = 1326.8 ***	F = 727.4 ***	F = 0.0 ns	F = 0.1 ns	F = 0.5 ns	F = 492.9 ***	F = 102.1 ***
B_1_: MS = 58963	B_1_: MS = 32	B_1_: MS = 0.0011	B_1_: MS = 1284.5	B_1_: MS = 66.7	B_1_: MS = 62,597	B_1_: MS = 10.11	B_1_: MS = 10.532
F = 12.1 ***	F = 1.5 ns	F = 1.5 ns	F = 3.6 ns	F = 0.1 ns	F = 8.8 **	F = 30.2 ***	F = 93.5 ***
C_1_: MS = 333	C_1_: MS = 10	C_1_: MS = 0.0008	C_1_: MS = 230.5	C_1_: MS = 1211.3	C_1_: MS = 8825	C_1_: MS = 9.48	C_1_: MS = 2.64
F = 0.1 ns	F = 0.4 ns	F = 1.0 ns	F = 0.6 ns	F = 2.8 ns	F = 1.2 ns	F = 28.3 ***	F = 23.4 ***
D_116_: MS = 4850	D_116_: MS = 20	D_116_: MS = 0.0007	D_116_: MS = 349.5	D_116_: MS = 422.9	D_116_: MS = 7034	D_116_: MS = 0.33	D_116_: MS = 0.113
**EL/EW**	**ES**	**NL**	**NW**	**NL/NW**	**NS**	**NS/ES**	
A_1_: MS = 0.272	A_1_: MS = 40732	A_1_: MS = 95.94	A_1_: MS = 8.560	A_1_: MS = 0.9133	A_1_: MS = 3708	A_1_: MS = 0.01668	
F = 566.3 ***	F = 276.5 ***	F = 968.6 ***	F = 388.7 ***	F = 427.9 ***	F = 800.3 ***	F = 201.9 ***	
B_1_: MS = 0.027	B_1_: MS = 8430	B_1_: MS = 5.57	B_1_: MS = 0.736	B_1_: MS = 0.0402	B_1_: MS = 230	B_1_: MS = 0.00018	
F = 56.2 ***	F = 57.2 ***	F = 56.2 ***	F = 33.4 ***	F = 18.8 ***	F = 49.7 ***	F = 2.2 ns	
C_1_: MS = 0.001	C_1_: MS = 3370	C_1_: MS = 3.09	C_1_: MS = 1.064	C_1_: MS = 0.0012	C_1_: MS = 188	C_1_: MS = 0.00064	
F = 2.2 ns	F = 22.8 ***	F = 31.1 ***	F = 48.3 ***	F = 0.579 ns	F = 40.6 ***	F = 7.7 **	
D_116_: MS = 0.0004	D_116_: MS = 147	D_116_: MS = 0.1	D_116_: MS = 0.022	D_116_: MS = 0.0021	D_116_: MS = 5	D_116_: MS = 0.00008	

Legend: RBC—erythrocyte count, Hb—hemoglobin concentration, PCV—packed cell volume (hematocrit value), MCH—mean corpuscular hemoglobin, MCHC—mean corpuscular hemoglobin concentration, MCV—mean corpuscular volume, EL—erythrocyte length, EW—erythrocyte width, ES—erythrocyte size, NL—nucleus length, NW—nucleus width, and dNS—nucleus size. Significance codes: ** *p* < 0.01; *** *p* < 0.001; ns *p* > 0.05.

**Table 3:** Descriptive statistics (mean ± SD; Min–Max) and results from the comparisons of the erythrogram and erythrocyte metric parameters of *Pelophylax ridibundus* individuals from two sites in southern Bulgaria.

Parameters	Reference Site: Vacha River, Near the Town of Krichim		Polluted Site: Sedimentation Lake of Brikel TPP		Tukey HSD (post hoc) Test
	Female (1), *n* = 30	Male (2), *n* = 30	Female (3), *n* = 30	Male (4), *n* = 30	
RBC (×10^6^/μL)	401 ± 45.44 (310–460)	360 ± 45.94 (300–460)	652.33 ± 87.8 (430–780)	604.67 ± 86.69 (460–770)	1/2 ns, 1 < 3 ***, 1 < 4 ***, 2 < 3 ***, 2 < 4 ***, 3 > 4 *
Hb (g/dL)	52.88 ± 3.03 (44.93–59.9)	51.28 ± 3.77 (45.21–65.28)	82.29 ± 5.22 (72.4–89.75)	81.83 ± 5.54 (64.07–89.85)	1 > 2 ns, 1 < 3 ***, 1 < 4 ***, 2 < 3 ***, 2 < 4 ***,3/4 ns
PCV (L/L)	0.24 ± 0.03 (0.2–0.31)	0.24 ± 0.03 (0.21–0.3)	0.38 ± 0.03 (0.32–0.43)	0.37 ± 0.03 (0.31–0.42)	1 > 2 ns, 1 < 3 ***, 1 < 4 ***, 2 < 3 ***, 2 < 4 ***, 3/4 ns
MCH (pg)	141.21 ± 16.42 (109.59–179.2)	144.99 ± 19.55 (105.17–176.67)	139.18 ± 17.16 (120.14–181.9)	148.5 ± 21.25 (117.63–196.38)	1/2 ns, 1/3 ns, 1/4 ns, 2/3 ns. 2/4 ns, 3/4 ns
MCHC (g/L)	222 ± 25.67 (149.77–275.1)	214.15 ± 26.76 (150.7–261.12)	217.27 ± 11.46 (185.38–241.54)	222.14 ± 13.62 (200.83–265.19)	1/2 ns, 1/3 ns, 1/4 ns, 2/3 ns, 2/4 ns, 3/4 ns
MCV (fL)	632.74 ± 70.8 (517.44–758.33)	695.57 ± 99.92 (521.74–983.33)	638.96 ± 69.85 (537.86–806.25)	667.49 ± 90.89 (528.57–870.75)	1 < 2 *, 1/3 ns 1/4 ns, 2 > 3 *, 2/4 ns, 3/4 ns
EL (μm)	24.23 ± 0.7 (22.7–25.88)	24.21 ± 0.43 (23.43–25.03)	22.45 ± 0.45 (21.58–23.45)	21.3 ± 0.67 (20.05–22.38)	1/2 ns, 1 > 3 ***,1 > 4 ***, 2 > 3 ***, 2 > 4 ***, 3 > 4 ***
EW (μm)	14.03 ± 0.44 (12.95–15.08)	13.73 ± 0.33 (13.08–14.18)	13.7 ± 0.28 (13.03–14.35)	12.81 ± 0.26 (12.4–13.25)	1 > 2 ***, 1 > 3 **, 1 > 4 ***, 2/3 ns, 2 > 4 ***, 3 > 4 **
EL/EW ratio	1.73 ± 0.02 (1.7–1.77)	1.77 ± 0.03 (1.73–1.85)	1.64 ± 0.02 (1.59–1.68)	1.66 ± 0.03 (1.61–1.72)	1 < 2 ***, 1 > 3 ***, 1 > 4 ***, 2 > 3 ***, 2 > 4 ***, 3<4 ***
ES (μm^2^)	268.46 ± 16.25 (231.34–309.11)	262.3 ± 10.71 (243.76–279.79)	242.21 ± 9.46 (222.84–264.66)	214.85 ± 11 (195.39–232.3)	1/2 ns, 1 > 3 ***, 1 > 4 ***, 2 > 3 ***, 2 > 4 ***, 3 > 4 ***
NL (μm)	9.65 ± 0.34 (9.03–10.58)	9.54 ± 0.26 (9.05–10.13)	8.18 ± 0.29 (7.58–8.9)	7.43 ± 0.36 (6.8–8)	1/2 ns, 1 > 3 ***, 1 > 4 ***, 2 > 3 ***, 2 > 4 ***, 3 > 4 ***
NW (μm)	5.52 ± 0.18 (5.18–5.8)	5.55 ± 0.17 (5.18–5.88)	5.17 ± 0.13 (4.9–5.55)	4.83 ± 0.11 (4.58–5.03)	1/2 ns, 1 > 3 ***, 1 > 4 ***, 2 > 3 ***, 2 > 4 ***, 3 > 4 ***
NL/NW ratio	1.76 ± 0.06 (1.65–1.89)	1.73 ± 0.04 (1.64–1.8)	1.59 ± 0.03 (1.53–1.66)	1.55 ± 0.05 (1.48–1.63)	1/2 ns, 1 > 3 ***, 1 > 4 ***, 2 > 3 ***, 2 > 4***, 3 > 4**
NS (μm^2^)	42.23 ± 2.47 (37.68–47.06)	41.96 ± 2.11 (37.37–46.43)	33.62 ± 2.01 (29.67–39.15)	28.34 ± 1.98 (24.55–31.77)	1/2 ns, 1 > 3 ***, 1 > 4 ***, 2 > 3 ***, 2 > 4 ***, 3 > 4 ***
NS/ES ratio	0.16 ± 0.01 (0.14–0.19)	0.16 ± 0.01 (0.15–0.17)	0.14 ± 0.01 (0.12–0.17)	0.13 ± 0.00 (0.12–0.14)	1/2 ns, 1 > 3 ***, 1 > 4 ***, 2 > 3 ***, 2 > 4 ***, 3 > 4 *

Legend: RBC—erythrocyte count, Hb—hemoglobin concentration, PCV—packed cell volume (hematocrit value), MCH—mean corpuscular hemoglobin, MCHC—mean corpuscular hemoglobin concentration, MCV—mean corpuscular volume, EL—erythrocyte length, EW—erythrocyte width, ES—erythrocyte size, NL—nucleus length, NW—nucleus width, NS—nucleus size, and n—number of individuals. The symbols < and > compare the mean values of the parameters. Significance codes: * *p* < 0.05; ** *p* < 0.01; *** *p* < 0.001; ns *p* > 0.05.

## Data Availability

The datasets used and/or analyzed during the current study are available from the corresponding author upon reasonable request.

## Abbreviations

The following abbreviations are used in this manuscript:

TPP	Thermal Power Plant
RS	Reference Site
PS	Polluted Site
EC	Electrical Conductivity
DO	Dissolved Oxygen
Ox	Oxygenation
BOD5	Biological Oxygen Demand Five Days
COD	Chemical Oxygen Demand
NH^4+^-N	Ammonium Nitrogen
NO_2_^−^-N	Nitrite Nitrogen
NO_3_^−^-N	Nitrate Nitrogen
TN	Total Nitrogen
PO_4_^3−^	Orthophosphate
TP	Total Phosphorus
SVL	Snout Vent Length
RBC	Red Blood Cell
Hb	Hemoglobin
PCV	Packed Cell Volume (Hematocrit)
MCH	Mean Corpuscular Hemoglobin
MCHC	Mean Corpuscular Hemoglobin Concentration
MCV	Mean Corpuscular Volume
EL	Erythrocyte Length
EW	Erythrocyte Width
ES	Erythrocyte Size
NL	Nucleus Length
NW	Nucleus Width
NS	Nucleus Size
KMO test	Kaiser–Meyer–Olkin Test
PCA	Principal Component Analysis
PC	Principal Component
ANOVA	Analysis of Variance
df	Degree of Freedom
HSD	Honestly Significant Difference
